# Brain Anatomy Alterations and Mental Health Challenges Correlate to Email Addiction Tendency

**DOI:** 10.3390/brainsci12101278

**Published:** 2022-09-22

**Authors:** Saeid Sadeghi, Hikaru Takeuchi, Bita Shalani, Yasuyuki Taki, Rui Nouchi, Ryoichi Yokoyama, Yuka Kotozaki, Seishu Nakagawa, Atsushi Sekiguchi, Kunio Iizuka, Sugiko Hanawa, Tsuyoshi Araki, Carlos Makoto Miyauchi, Kohei Sakaki, Takayuki Nozawa, Shigeyuki Ikeda, Susumu Yokota, Daniele Magistro, Yuko Sassa, Ryuta Kawashima

**Affiliations:** 1 Institute for Cognitive and Brain Sciences (ICBS), Shahid Beheshti University, Tehran 19839-69411, Iran; 2 Division of Developmental Cognitive Neuroscience, Institute of Development, Aging and Cancer, Tohoku University, Sendai 980-8575, Japan; 3 Center of Excellence in Cognitive Neuropsychology, Shahid Beheshti University, Tehran 19839-69411, Iran; 4 Department of Psychology, Faculty of Humanities, Tarbiat Modares University, Tehran 14117-13116, Iran; 5 Division of Medical Neuroimaging Analysis, Department of Community Medical Supports, Tohoku Medical Megabank Organization, Tohoku University, Sendai 980-8575, Japan; 6 Department of Radiology and Nuclear Medicine, Institute of Development, Aging and Cancer, Tohoku University, Sendai 980-8575, Japan; 7 Creative Interdisciplinary Research Division, Frontier Research Institute for Interdisciplinary Science, Tohoku University, Sendai 980-8575, Japan; 8 Human and Social Response Research Division, International Research Institute of Disaster Science, Tohoku University, Sendai 980-8575, Japan; 9 Department of Advanced Brain Science, Institute of Development, Aging and Cancer, Tohoku University, Sendai 980-8575, Japan; 10 School of Medicine, Kobe University, Kobe 657-8501, Japan; 11 Division of Clinical Research, Medical-Industry Translational Research Center, School of Medicine, Fukushima Medical University, Fukushima 960-1295, Japan; 12 Department of Human Brain Science, Institute of Development, Aging and Cancer, Tohoku University, Sendai 980-8575, Japan; 13 Department of Psychiatry, Tohoku Medical and Pharmaceutical University, Sendai 980-8575, Japan; 14 Department of Behavioral Medicine, National Institute of Mental Health, National Center of Neurology and Psychiatry, Tokyo 153-0051, Japan; 15 Department of Psychiatry, Tohoku University Graduate School of Medicine, Sendai 980-8575, Japan; 16 Advantage Risk Management Co., Ltd., Tokyo 153-0051, Japan; 17 Research Center for the Earth Inclusive Sensing Empathizing with Silent Voices, Tokyo Institute of Technology, Tokyo 152-8552, Japan; 18 Department of General Systems Studies, Graduate School of Arts and Sciences, The University of Tokyo, Tokyo 153-0051, Japan; 19 Department of Ubiquitous Sensing, Institute of Development, Aging and Cancer, Tohoku University, Sendai 980-8575, Japan; 20 Division for Experimental Natural Science, Faculty of Arts and Science, Kyushu University, Fukuoka 819-0395, Japan; 21 Department of Sport Science, School of Science and Technology, Nottingham Trent University, Nottingham NG1 4FQ, UK

**Keywords:** email addiction tendency, brain structures, depression, reasoning

## Abstract

Despite the widespread use of email, our knowledge regarding the consequences of email addiction is lacking. The purpose of this study was to develop an email addiction tendency scale to evaluate its correlation to behavior and brain structure. Following this, the validity and reliability of the developed scale was investigated. We used voxel-based morphometry, correlation, and univariate regression analysis to assess the relationships between email addiction tendency scores and regional gray and white matter volumes, depression, and nonverbal reasoning abilities in a large sample of healthy young adults (n = 1152; mean age, 20.69 ± 1.84 years). The content validity ratio, content validity index, principal component analysis, and confirmatory factorial analysis all showed that the email addiction tendency scale (EATS) has high validity. Additionally, the Cronbach’s alpha internal consistency and split-half reliability coefficient showed that the EATS has high reliability. We found that email addiction tendency scores were significantly negatively correlated with nonverbal reasoning. We also observed that the email addiction tendency scores were significantly and positively correlated with depression symptom severity and gray matter volume of the left rostrolateral prefrontal cortex (RLPC) in subjects. These results indicate that email addiction tendency is associated with lower mental health outcomes and increased GMV in the left RLPC.

## 1. Introduction

In recent years, access to digital devices has increased dramatically. Indeed, it is estimated that there will be approximately 3.01 billion smartphone users worldwide by 2021 [1]. Most internet users use social media applications several times a day, and can do so anytime and anywhere [2]. One of the most popular functions used on digital devices is online-communication applications.

The last report showed that 58.4% of the worldwide population uses social media every day and spends about 2 h and 27 min on it [3]. Accumulating evidence shows that the repeated use of social media can lead to habitual usage and impulsive responses to social media cues, as well as behavioral dependence [4,5]. To define dependence in terms of substance abuse, the Diagnostic and Statistical Manual of Mental Disorders (DSM 5) uses two concepts: behavioral and physiological [6]. In behavioral dependence, substance-seeking behavior and pathological use patterns are emphasized, while physical dependence refers to the physical effects of repeated episodes of substance abuse. Tolerance or withdrawal are associated with definitions that emphasize physical dependence, and the term addiction is nearly the same concept as physical dependence.

So, behavioral addiction to technology, such as email addiction and mobile phone addiction, can be defined as excessive use and intermittent cravings [7]. As defined by LaRose, Mastro [8] and Thadani and Cheung [9], dependence on social networks is the inability to control the way one uses the tool, which may negatively affect one’s personal, family, and professional lives. Social media addiction is the compulsive use of social networking sites and apps, which manifests as symptoms of behavioral dependence [10,11].

Previous studies have shown that excessive use of social networks is associated with mental health problems in users [12,13,14]. For example, Donnelly and Kuss [15] showed that social network addiction is related to depression in young adults. Similarly, McDougall, Walsh [16] revealed that daily use of social networking is significantly correlated with depression. Overall, a systematic review with meta-analysis studies demonstrated that social network dependence is associated with psychiatric disorders and symptoms such as poor sleep quality, anxiety, stress, depression, and attention-deficit/hyperactivity disorder [17,18,19]. Moreover, within the last decade, several studies have been conducted that characterize the impacts of social network system addiction on users’ cognitive functions. These studies reported that several key cognitive domains, such as information processing, executive control, reward processing, and socio-cognitive functions, have been affected by internet-related technologies [20]. Additional studies have shown that substance and alcohol abuse are associated with impaired cognitive abilities (e.g., speed processing, abstract reasoning, planning, inhibition, working and long-term memory, sustained attention, and cognitive flexibility [21,22,23,24,25]). However, the relationships between email addiction and users’ mental health and cognitive abilities have not been thoroughly studied.

The neural correlates of internet addiction have been studied much more than the neural correlates of social networks addiction. In studies using magnetic resonance imaging (MRI), internet addiction scores significantly correlate with GMVs in the right-middle frontal gyrus, supplementary motor area (SMA), anterior cingulate cortex (ACC), left rostral anterior cingulate cortex (rACC), orbitofrontal cortex (OFC), bilateral dorsolateral prefrontal cortex (DLPFC), cerebellum, right supramarginal gyrus, and the post-central gyrus (postCG) [26,27,28,29]. There have been few studies examining the effects of social networking on brain anatomy. He and Turel [30] found that social network site dependence scores are negatively correlated with gray matter volume (GMV) in the bilateral amygdala. Meanwhile, the same scores are positively correlated with GMV in the anterior/mid-cingulate cortex. The ACC and amygdala are thought to be key neural substrates for social information processing and emotional responses [31,32]. In another study, a research team showed that the GMVs of the posterior parts of the bilateral middle, superior temporal, and left fusiform gyri are positively associated with the level of Facebook use [33]. The superior temporal cortex and fusiform gyrus are thought to be involved in the ability to recognize faces and visual analysis of social information [34,35]. The brain structures linked to email dependence have not been studied to the best of our knowledge.

Also, while internet addiction has been extensively examined over the years [36,37,38,39], very few studies have developed or validated tools to measure and confirm the reality of social media addiction [40]. Specifically, significant research into email and its impact on our lives is lacking. Nowadays, checking the email is an inevitable part of our daily activities. Renaud and Ramsay [41] reported that some individuals check their email inbox 30 to 40 times an hour. However, no reliable tool exists for measuring the tendency for email addiction. Therefore, the first aim of this study was to develop a highly valid and reliable email addiction tendency scale (EATS). The second objective was to identify the relationships between email addiction tendency and behavioral outcomes and brain structures.

On the basis of the previous studies, we hypothesized that higher EATS scores may be associated with structural alterations in the frontal and temporal areas known to contribute to dependence vulnerability [29,42,43,44,45]. Furthermore, we hypothesized that email addiction tendency is significantly positively correlated with the severity of depression symptoms and significantly negatively correlated with nonverbal reasoning abilities.

## 2. Materials and Methods

### 2.1. Participants and Procedures

The subjects of this study were 1152 healthy right-handed young adults (666 males, mean age 20.79 years, SD = 1.89 years and 486 females, mean age 20.60 years, SD = 1.61 years). This study is part of an ongoing project to investigate the associations among brain imaging characteristics, cognitive functions, aging, genetics, and daily habits. All subjects were students from the Tohoku University or neighboring universities and colleges in Japan. All had normal vision, and none had a history of neurological or psychiatric illness. None reported the currently using psychoactive or other drugs that could negatively impact cognitive ability. As part of our laboratory’s routine questionnaire, each subject was asked about their current or previous experiences with any of the listed diseases and drugs that they had recently taken, as well as their history of psychiatric and neurological diseases and/or recent drug use. In addition, the questionnaire required personal contact information, their age, their birthday, their institutes, and their weight, height, and sex. We used the Edinburgh Handedness Inventory to evaluate handedness in subjects [46]. A number of previous studies have demonstrated significant differences between righthanders and lefthanders in terms of brain morphology and activity patterns [47,48,49,50,51]. In this context, left-handed individuals tend to be excluded from fMRI studies. The Ethics Committee of the Tohoku University approved all procedures. These were performed in accordance with relevant guidelines and regulations. Written, informed consent was obtained from each subject for all projects in which they participated. Descriptions for this subsection are adapted from a previous study that used similar methods [52].

For scale construction and validation, we developed the email addiction tendency scale. The content domain of the construct that the scale is designed to measure was identified by literature review on internet-related technology addiction tools. We developed the EATS based on the Young’s internet addiction tendence (IAT) scale [39]. Furthermore, an expert panel evaluated the validity of the scale. The determination of the number of experts has always been partly arbitrary. A minimum of five people is recommended to have sufficient control over chance agreement [53]. Our expert panel consisted of professionals with research experience or work in the field and had doctorate degrees in psychology.

### 2.2. Psychological Assessments

#### 2.2.1. EATS

To assess email addiction tendency, we developed the EATS. The EATS tool consists of 20 questions answered on a 1–5 scale from 1 = “Never” to 5 = “always.” The scale is self-administered and requires 5–10 min for completion. The EATS scale minimum and maximum scores are 20 and 100, respectively. Higher scores reflect a greater tendency toward email addiction. We calculated and reported the psychometric properties of the EATS in this study.

#### 2.2.2. Internet Addiction Tendency (IAT)

The IAT tool [39] consists of 20 items, with answers ranging from 1 = “rarely” to 5 = “always”. The scale is self-administered and takes 5 to 10 min to complete. IAT measures the impact of internet use on daily life, social life, productivity, sleep patterns, and people’s feelings. The minimum and maximum values on the IAT scale are 20 and 100, respectively. The higher the value, the greater the tendency for internet addiction. The Japanese version of this scale has demonstrated high reliability and validity [54]. We used the Japanese version of Young’s IAT scale to assess the EATS’s convergent validity.

#### 2.2.3. Raven’s Advanced Progressive Matrices

Raven’s advanced progressive matrices were used to measure the general nonverbal intelligence and abstract reasoning ability of the participants [55]. Raven’s advanced progressive matrices are constructed for those with a higher level of intelligence, such as students pursuing advanced scientific or technical studies. The reliability and validity of this tool has been demonstrated across a range of populations [56].

#### 2.2.4. Beck Depression Inventory-Second Version (BDI-II)

The BDI-II was used to assess the severity of depression symptoms in participants [57]. The reliability and validity of the BDI-II has been demonstrated across a range of populations, including college students [58].

### 2.3. Brain IMAGE Acquisition

The MRI acquisition methods are described in our previous study [59]. Briefly, all MRI data were acquired using a 3T Philips Achieva scanner. Diffusion-weighted data were acquired using a spin-echo EPI sequence (TR = 10,293 ms, TE = 55 ms, FOV = 22.4 cm, 2 × 2 × 2 mm^3^ voxels, 60 slices, SENSE reduction factor = 2, number of acquisitions = 1).

### 2.4. Statistical Analysis

The psychometric properties of the EATS were analyzed. We calculated the index of content validity (CVI) and the content validity ratio (CVR) to assess content validity [60]. We used the following two kinds of CVI: Item-CVI (I-CVI) and Scale-level CVI (S-CVI).

Additionally, principal component analysis (PCA) and confirmatory factorial analysis (CFA) were used to assess scale construct validity. Furthermore, the Cronbach’s alpha, split-half reliability coefficient, and item–total correlation were determined to assess reliability.

Quality control of the MRI images has been conducted by visual inspection and images with artifacts had been removed from the images. Preprocessing of the neuroimaging data was performed using Statistical Parametric Mapping software (SPM12; Wellcome Department of Cognitive Neurology, London, UK) and implemented in MATLAB (Mathworks, Inc., Natick, MA, USA). For analysis, T1-weighted structural images of each individual were segmented using the new segmentation algorithm implemented in SPM12. These were further normalized to the Montreal Neurological Institute (MNI) space-to-yield images with 1.5 × 1.5 × 1.5 mm^3^ voxels, using diffeomorphic anatomical registration through an exponentiated lie algebra registration process implemented in SPM12. We also performed a volume change correction (modulation) [61]. Subsequently, the generated regional GMV (rGMV) and white matter volume (rWMV) images were smoothed by convolution using an isotropic Gaussian kernel of 8-mm full width at half maximum. These procedures were adapted from our previous study using similar methods. For full descriptions of these procedures, see our previous work [52].

Statistical analyses of imaging data were performed with SPM8. Structural whole-brain regression analyses were performed to investigate any associations between email dependence scores and rGMV and rWMV. Age, sex, and total intracranial volume, calculated using voxel-based morphometry (for details of calculation see [62]), were added as covariates. Multiple comparison correction was performed using threshold-free cluster enhancement (TFCE) [63] with randomized (5000 permutations) nonparametric testing, using the TFCE toolbox (http://dbm.neuro.uni-jena.de/tfce/ accessed on 15 August 2019). We applied a threshold of family-wise error correction at *p* < 0.05. SPM8 was used for this analysis because of its better compatibility with TFCE software and our in-house scripts [64].

## 3. Results

The characteristics of the psychological variables of participants are presented in Table 1.

### 3.1. Scale Validation Results

#### 3.1.1. Validity

##### Content Validity

All items with an I-CVI of 1.00, S-CVI/UA = 1, and S-CVI/Ave = 1 were considered relevant. The UA is calculated by adding all I-CVI values equal to 1.00 (20 items) divided by 20, while the average takes the sum of all I-CVI values (20) divided by 20. Overall, the UA and the Average demonstrate high content validity for the EATS.

Furthermore, the CVR was generated for each item. Items that were marked essential had a CVR of >0.99 (this value is based on the total number of experts, N = 5, and the numerical values of the Lawshe table) [65]. Useful but not essential items can be eliminated. However, in this case, they were modified (items: 2, 4, 10, and 11). The average CVR value was 0.83.

##### Construct Validity

**Figure 1:**
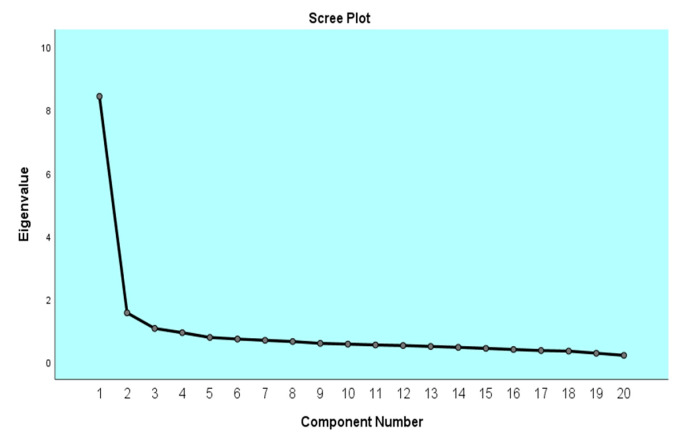
Scree plot to examine the number of EATS factors.

PCA was used to assess the construct validity of the EATS [66]. The Kaiser–Meyer–Olkin assessment of sampling adequacy of the 20 items was 0.945, which is well above the commonly acceptable limit of 0.600. Bartlett’s test of sphericity that evaluates the appropriateness of factoring also indicated that the identity matrix was significant (χ^2^: 11,380.66, df = 190, *p* < 0.0001). The Scree plot results proposed a one-factor solution. From the original 20-item set, no items were removed because the factor loading of the items was higher than 0.30 and all items fit well into the factor (Figure 1).

All of the 20 items loaded on the main factor and the main factor of the final solution cumulatively accounted for 42.151% of the variance. The results of the PCA are shown in Table 2.

**Figure 2:**
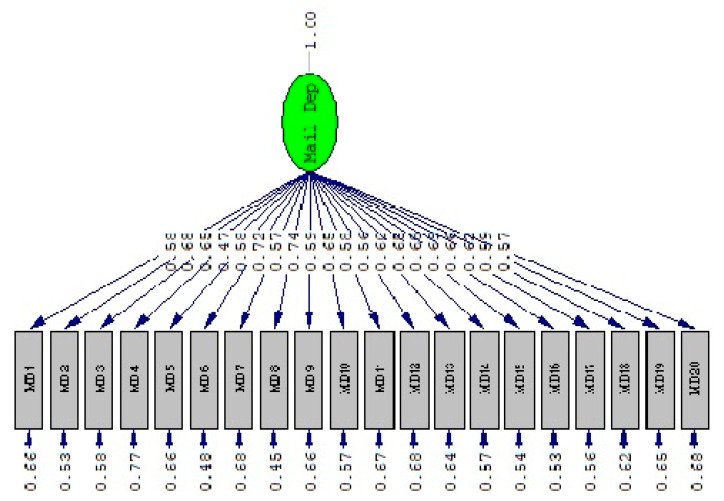
CFA for email addiction tendency scale.

In the next step, a CFA was used to recognize the EATS’s factor structure that was confirmed in the EFA. The goodness of fit model indices were calculated as follows: comparative fit index = 0.94, normed fit index = 0.93, goodness of fit index = 0.80, relative fit index = 0.93, and incremental fit index = 0.94. According to these indices, the construct is considered acceptable [67,68]. Furthermore, the CFA results in our current approach showed that the model is coherent. As presented in Figure 2, all items loaded on the factor are statistically significant (*p* < 0.05).

#### 3.1.2. Reliability

The internal reliability of the scale was calculated using Cronbach’s alpha, split-half reliability coefficient, and item–total correlation. The Cronbach’s alpha internal consistency score and split-half reliability coefficient of the scale were 0.916 and 0.843, respectively. The item–total correlation coefficients ranged from 0.52 to 0.75 for the 20 items. All correlation coefficients were positive, as well as statistically significant.

### 3.2. Behavioral Results

**Figure 3:**
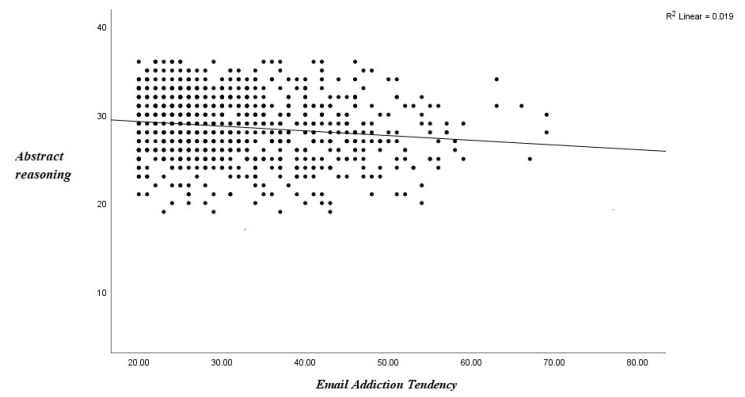
Associations between email dependence and abstract reasoning.

**Figure 4:**
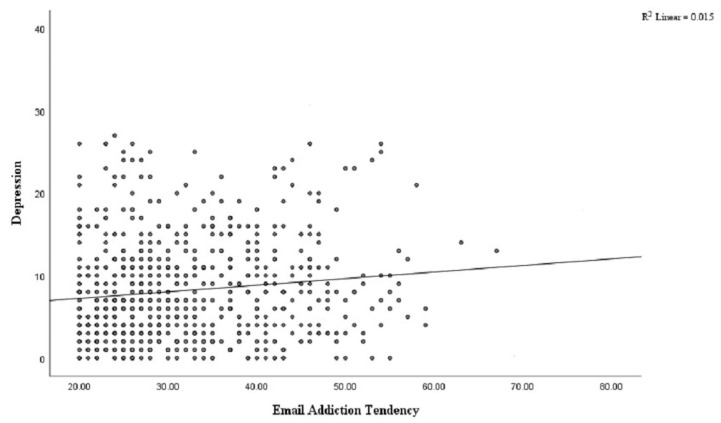
Associations between email addiction tendency and depression severity.

Univariate regression analyses were employed to assess the relationship between email addiction tendency, nonverbal reasoning, and depression in subjects. The results revealed that email addiction tendency was significantly negatively correlated with nonverbal reasoning (Figure 3. *r* = −0.14, *p* < 0.01). Email addiction tendency was also significantly positively correlated with depression symptom severity (Figure 4. *r* = 0.12, *p* < 0.01) and internet addiction tendencies (*r* = 0.30, *p* < 0.01) in subjects. Regression analyses showed that for each unit of increase in the EATS scores, the nonverbal reasoning decreased by 0.019 (t = −3.89, *p* < 0.0001), and depression severity increased by 0.015 (t = 3.22, *p* < 0.001).

### 3.3. Neuroimaging Results

**Figure 5:**
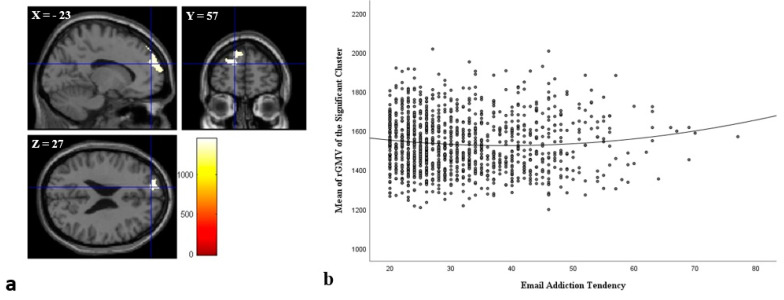
Gray matter brain regions correlate to email addiction tendency scores in young adults. Regions with significant correlation between email addiction tendency scores and GMVs are overlaid on a “single subject” T1-weighted image from SPM8. Results were obtained using a threshold of TFCE with *p* < 0.05 based on 5000 permutations (**a**). A significant, positive correlation was found in the left rostrolateral prefrontal cortex (**b**).

Voxel-based morphometry analysis revealed a significant positive correlation between the GMV of the left rostrolateral prefrontal cortex (RLPC) and email addiction tendency scores (Table 3 and Figure 5).

## 4. Discussion

To the best of our knowledge, this is the first study to not only develop and validate a scale that measures email addiction, but also to investigate its behavioral and neurological correlations. Our findings confirm our hypothesis: email addiction tendency is correlated with mental health issues, as well as changes in brain structures involvedin high-level cognitive functioning.

First, the I-CVI, S-CVI/UA, S-CVI/Ave, and average CVR demonstrated the high content validity of the EATS. The PCA results showed the one-factor structure of the scale and the CFA supported the scale construct validity. Moreover, the Cronbach’s alpha and the Guttman split-half coefficients revealed that the EATS is highly reliable. The EATS provides a unique self-reporting tool to assess an individual’s dependence on email. Since it is well established that excessive use of internet is problematic [36,37,69,70], it is assumed that the EATS can evaluate email addiction tendency as a behavioral addiction.

Although this study shows the one-factor structure of EATS, like Young’s IAT [39,71], the results revealed that the EATS correlates moderately with Young’s IAT score (*r* = 0.30). This finding establishes that although internet addiction tendency and email addiction tendency have a positive and significant relationship with each other, they are, in fact, two independent constructs. The concepts of internet addiction and social network addiction have not been well-defined, largely because there are no gold standards for measuring these conditions, nor is there any widely accepted theory [72,73,74]. However, it is thought that email addiction is a subtype of internet addiction. As an example, Griffiths, Kuss [11] proposes three subtypes of internet addicts, based on the “object” of the addiction: online games, sex, and email or text messages. Social networks are an online activity where texting or emails are predominant, despite their being used for game-playing and even sex-related purposes.

Social network addiction has been associated with mental health problems in previous studies [15,36,75,76]. Interestingly, our results show that subjects who are more dependent on email experience more symptoms of depression and exhibit more deficits in nonverbal reasoning ability. These findings are in agreement with previous studies, demonstrating that problematic social network usage is associated with psychiatric disorder symptoms [17,18,19]. Li and Mo [77], in a large-scale prospective cohort study in Chinese adolescents, showed that the addictive use of online social networks is accompanied by an increase in the level of depression symptoms. One explanation for this finding is that the excessive use of online social networking displaces daily time spent with peers and family, leading to the withdrawal from interpersonal offline activities. This, in turn, increases negative emotions, such as depression symptoms [78]. In line with our findings, a recent study by Raj [79] has shown that internet-addicted subjects have more problems with abstract reasoning than nonaddicts. Moreover, studies by Romero-Martinez, Vitoria-Estruch [21] and Bagga andSingh [23] have shown impairments in abstract reasoning ability in alcohol-dependent subjects. Our findings suggest that email dependence has a common cognitive underpinning with other addictions, including alcoholism.

This study also sought to expand our current understanding of the neuropsychological basis of email dependence using structural MRI. The MRI results showed that email addiction tendency was significantly and positively correlated with the GMV of the left RLPC. Previous studies showed that the rostral areas of the lateral prefrontal cortex play an important role in higher levels of cognitive control and abstract representations [80,81,82,83]. One study by Nee and D’Esposito [84] showed that the rostral areas of the lateral prefrontal cortex are correlated with abstract processing, organizing behavior according to future considerations (e.g., goals or plans), and future-oriented processing. These results confirmed our hypothesis that email addiction tendency might be correlated with changes in brain regions implicated in higher cognitive functions such as cognitive control. Cognitive control deficits and impulsivity play critical roles in daily life and are associated with influential behavioral styles and problems such as driving violations [85], drinking [86], eating disorders [87], and substance addiction [88].

This study had two limitations. First, the cross-sectional design excludes the establishment of a causal relationship between email addiction tendency, mental health problems, and changes in specific brain structures. Second, because the study cohort consisted of only healthy, young people with relatively high levels of education, these results cannot be extrapolated to the general population.

## 5. Conclusions

In the present study, we developed a scale to assess the aspects of email addiction in young adults that affect mental health and brain structures of users, either directly or indirectly. Our findings show that our EATS provides a simple and quick method for evaluating email addiction tendency in young adults (Appendix A). The EATS had good validity and reliability. We also demonstrated that the increased tendency for email addiction is associated with more symptoms of depression and impairments in abstract reasoning ability. Moreover, email addiction tendency was characterized by increased GMV in the left RLPC brain region. Previous studies have also shown that the RLPC has a significant relationship with cognitive control.

## Figures and Tables

**Table 1:** Characteristics of the psychological variables of the subjects.

Variables	Descriptive Statistics		
	Sex	M	SD
**Email addiction tendency**	Male	31.60	10.05
	Female	32.92	9.64
**Internet addiction tendency**	Male	41.32	13.10
	Female	38.62	12.58
**Nonverbal reasoning**	Male	28.91	3.75
	Female	28.20	3.81
**Depression**	Male	8.05	6.20
	Female	8.35	6.50

**Abbreviations**: M, mean; SD, standard deviation.

**Table 2:** Results of PCA of the EATS.

No.	Items	Factor Loading
1	Have you ever emailed longer than you expected?	0.605
2	Have you neglected to study because you spent a lot of time exchanging emails?	0.698
3	Do you ever choose to spend more time with the stimulus you get by email rather than making friends with your friends?	0.678
4	Do you ever make new acquaintances by email?	0.507
5	Have anyone around you complained about the time you spend exchanging emails?	0.607
6	Is the time spent on email negatively impacting school grades and study?	0.727
7	Do you want to check your email when you have other things to do?	0.593
8	Does email have a negative impact on study efficiency and outcomes?	0.745
9	When asked what you are doing by email, do you defend yourself or become secretive?	0.614
10	Do you ever try keeping real life problems out of your mind, thinking about having fun with email?	0.681
11	Are you conscious of yourself looking forward to the next email?	0.610
12	Do you ever feel anxious that life without email would be boring, empty, and dreadful?	0.597
13	If someone interrupts you while you are emailing, do you bluntly say back, scream, or get annoyed?	0.632
14	Can I get sleep deprived because I email at midnight?	0.674
15	Do you think about email when you are not emailing, or fantasize about emailing?	0.710
16	Do you make excuses for “only a few minutes left” when you are emailing?	0.707
17	Have you ever tried shortening your email time and failed?	0.676
18	Do you want to hide from people how long you have been emailing?	0.645
19	Have you ever opted to spend more time exchanging emails instead of going out with others?	0.626
20	If you don’t have a new email, you will feel depressed, in a bad mood, and frustrated, but have you ever experienced that you can get rid of it immediately by emailing?	0.602
*Percentage of total variance explained*		**42.15**

**Table 3:** Brain gray matter regions that have a significant positive correlation with email addiction tendency.

Lobe (L/R)	Nearest GM Area	MNI Coordinate			TFCE Value	Corrected *p*-Value (FWE)	Cluster Size (mm^3^)
		X	Y	Z			
**Frontal** **(L)**	rostrolateral prefrontal cortex	−23	57	27	1451.63	0.031	3125

**Abbreviations**: GM, gray matter; L, left; R, right.

## Data Availability

All the experimental data obtained in the experiment of this study will be available to ones that were admitted in the ethics committee of Tohoku University, school of medicine. All the data sharing should be first admitted by the ethics committee of Tohoku University, school of medicine.

## Appendix A. Email Addiction Tendency Scale (EATS)

Instructions: Below is a list of questions that relate to life experiences common among people who show signs of problematic email checking. Please read each question carefully and indicate how often you have experienced the same or similar challenges in the past few months.

**Table:**

No.	Email Addiction Tendency Scale (EATS)					
	Please Mark the Appropriate Frequency for the Following 20 Questions about Emails Using Your PC or Mobile Phone.	Never 1	Rarely 2	Sometimes 3	Often 4	Always 5
1	Have you ever emailed longer than you expected?					
2	Have you neglected to study because you spent a lot of time exchanging emails?					
3	Do you ever choose to spend more time with the stimulus you get by email rather than making friends with your friends?					
4	Do you ever make new acquaintances by email?					
5	Have anyone around you complained about the time you spend exchanging emails?					
6	Is the time spent on email negatively impacting school grades and study?					
7	Do you want to check your email when you have other things to do?					
8	Does email have a negative impact on study efficiency and outcomes?					
9	When asked what you are doing by email, do you defend yourself or become secretive?					
10	Do you ever try keeping real life problems out of your mind, thinking about having fun with email?					
11	Are you conscious of yourself looking forward to the next email?					
12	Do you ever feel anxious that life without email would be boring, empty, and dreadful?					
13	If someone interrupts you while you are emailing, do you bluntly say back, scream, or get annoyed?					
14	Can I get sleep deprived because I email at midnight?					
15	Do you think about email when you are not emailing, or fantasize about emailing?					
16	Do you make excuses for “only a few minutes left” when you are emailing?					
17	Have you ever tried shortening your email time and failed?					
18	Do you want to hide from people how long you have been emailing?					
19	Have you ever opted to spend more time exchanging emails instead of going out with others?					
20	If you don’t have a new email, you will feel depressed, in a bad mood, and frustrated, but have you ever experienced that you can get rid of it immediately by emailing?					

## References

[1] Statista Prognose zur Anzahl der Smartphone-Nutzer Weltweit von 2012 bis 2021 (in Milliarden). https://de.statista.com/statistik/daten/studie/309656/umfrage/prognose-zur-anzahl-der-smartphone-nutzer-weltweit/.

[2] Elhai J.D., Rozgonjuk D., Alghraibeh A.M., Yang H. (2019). Disrupted daily activities from interruptive smartphone notifications: Relations with depression and anxiety severity and the mediating role of boredom proneness. Soc. Sci. Comput. Rev..

[3] (2022). Global Social Media Statistics Research Summary. https://www.smartinsights.com/social-media-marketing/social-media-strategy/new-global-social-media-research/#:~:text=More%20than%20half%20of%20the,within%20the%20last%2012%20months.

[4] Oulasvirta A., Rattenbury T., Ma L., Raita E. (2012). Habits make smartphone use more pervasive. Pers. Ubiquitous Comput..

[5] Van Deursen A.J., Bolle C.L., Hegner S.M., Kommers P.A. (2015). Modeling habitual and addictive smartphone behavior: The role of smartphone usage types, emotional intelligence, social stress, self-regulation, age, and gender. Comput. Hum. Behav..

[6] American Psychiatric Association (2013). Diagnostic and Statistical Manual of Mental Disorders (DSM-5®).

[7] Ezoe S., Toda M. (2013). Relationships of loneliness and mobile phone dependence with Internet addiction in Japanese medical students. Open J. Prev. Med..

[8] LaRose R., Mastro D., Eastin M.S. (2001). Understanding Internet usage: A social-cognitive approach to uses and gratifications. Soc. Sci. Comput. Rev..

[9] Thadani D.R., Cheung C.M. Online social network dependency: Theoretical development and testing of competing models. Proceedings of the 2011 44th Hawaii International Conference on System Sciences.

[10] Griffiths M. (2005). A ‘components’ model of addiction within a biopsychosocial framework. J. Subst. Use.

[11] Griffiths M.D., Kuss D.J., Demetrovics Z. (2014). Social networking addiction: An overview of preliminary findings. Behav. Addict..

[12] Chen A. (2019). From attachment to addiction: The mediating role of need satisfaction on social networking sites. Comput. Hum. Behav..

[13] Seabrook E.M., Kern M.L., Rickard N.S. (2016). Social networking sites, depression, and anxiety: A systematic review. JMIR Ment. Health.

[14] Marttila E., Koivula A., Räsänen P. (2021). Does excessive social media use decrease subjective well-being? A longitudinal analysis of the relationship between problematic use, loneliness and life satisfaction. Telemat. Inform..

[15] Donnelly E., Kuss D. (2016). Depression among users of social networking sites (SNSs): The role of SNS addiction and increased usage. J. Addict. Prev. Med..

[16] McDougall M.A., Walsh M., Wattier K., Knigge R., Miller L., Stevermer M., Fogas B.S. (2016). The effect of social networking sites on the relationship between perceived social support and depression. Psychiatry Res..

[17] Hussain Z., Griffiths M.D. (2021). The associations between problematic social networking site use and sleep quality, attention-deficit hyperactivity disorder, depression, anxiety and stress. Int. J. Ment. Health Addict..

[18] Piteo E.M., Ward K. (2020). Social networking sites and associations with depressive and anxiety symptoms in children and adolescents–a systematic review. Child Adolesc. Ment. Health.

[19] Vahedi Z., Zannella L. (2021). The association between self-reported depressive symptoms and the use of social networking sites (SNS): A meta-analysis. Curr. Psychol..

[20] Loh K.K., Kanai R. (2016). How Has the Internet Reshaped Human Cognition?. Neuroscientist.

[21] Romero-Martinez A., Vitoria-Estruch S., Moya-Albiol L. (2020). Cognitive profile of long-term abstinent alcoholics in comparison with non-alcoholics. Adicciones.

[22] Kornreich C., Delle-Vigne D., Knittel J., Nerincx A., Campanella S., Noel X., Hanak C., Verbanck P., Ermer E. (2011). Impaired conditional reasoning in alcoholics: A negative impact on social interactions and risky behaviors?. Addiction.

[23] Bagga D., Singh N., Singh S., Modi S., Kumar P., Bhattacharya D., Garg M.L., Khushu S. (2014). Assessment of abstract reasoning abilities in alcohol-dependent subjects: An fMRI study. Neuroradiology.

[24] Stein M., Steiner L., Fey W., Conring F., Rieger K., Federspiel A., Moggi F. (2021). Alcohol-related context modulates neural correlates of inhibitory control in alcohol dependent patients: Preliminary data from an fMRI study using an alcohol-related Go/NoGo-task. Behav. Brain Res..

[25] Mahoney J.J. (2019). Cognitive dysfunction in individuals with cocaine use disorder: Potential moderating factors and pharmacological treatments. Exp. Clin. Psychopharmacol..

[26] Pan N., Yang Y., Du X., Qi X., Du G., Zhang Y., Li X., Zhang Q. (2018). Brain structures associated with internet addiction tendency in adolescent online game players. Front. Psychiatry.

[27] Yuan K., Qin W., Wang G., Zeng F., Zhao L., Yang X., Liu P., Liu J., Sun J., von Deneen K.M. (2011). Microstructure abnormalities in adolescents with internet addiction disorder. PLoS ONE.

[28] Wang H., Jin C., Yuan K., Shakir T.M., Mao C., Niu X., Niu C., Guo L., Zhang M. (2015). The alteration of gray matter volume and cognitive control in adolescents with internet gaming disorder. Front. Behav. Neurosci..

[29] Sadeghi S., Takeuchi H., Shalani B., Taki Y., Nouchi R., Yokoyama R., Kotozaki Y., Nakagawa S., Sekiguchi A., Iizuka K. (2021). Brain structures and activity during a working memory task associated with internet addiction tendency in young adults: A large sample study. PLoS ONE.

[30] He Q., Turel O., Bechara A. (2017). Brain anatomy alterations associated with Social Networking Site (SNS) addiction. Sci. Rep..

[31] Gao Y.-J., Ren W.-H., Zhang Y.-Q., Zhao Z.-Q. (2004). Contributions of the anterior cingulate cortex and amygdala to pain-and fear-conditioned place avoidance in rats. Pain.

[32] Stone V.E., Baron-Cohen S., Calder A., Keane J., Young A. (2003). Acquired theory of mind impairments in individuals with bilateral amygdala lesions. Neuropsychologia.

[33] Turel O., He Q., Brevers D., Bechara A. (2018). Social networking sites use and the morphology of a social-semantic brain network. Soc. Neurosci..

[34] Buxbaum J., Hof P.R. (2012). The Neuroscience of Autism Spectrum Disorders.

[35] Stigler K.A., McDougle C.J., Buxbaum J., Hof P. (2013). Structural and functional MRI studies of autism spectrum disorders. The Neuroscience of Autism Spectrum Disorders.

[36] Kuss D.J., Lopez-Fernandez O. (2016). Internet addiction and problematic Internet use: A systematic review of clinical research. World J. Psychiatry.

[37] Pan Y.-C., Chiu Y.-C., Lin Y.-H. (2020). Systematic review and meta-analysis of epidemiology of internet addiction. Neurosci. Biobehav. Rev..

[38] Saletti S.M.R., Van den Broucke S., Chau C. (2021). The effectiveness of prevention programs for problematic Internet use in adolescents and youths: A systematic review and meta-analysis. Cyberpsychol. J. Psychosoc. Res. Cyberspace.

[39] Young K.S. (1998). Caught in the Net: How to Recognize the Signs of Internet Addiction--and a Winning Strategy for Recovery.

[40] Marulanda-Carter L., Jackson T.W. (2012). Effects of e-mail addiction and interruptions on employees. J. Syst. Inf. Technol..

[41] Renaud K., Ramsay J., Hair M. (2006). “You’ve got e-mail!”... shall I deal with it now? Electronic mail from the recipient’s perspective. Int. J. Hum.-Comput. Interact..

[42] Morein-Zamir S., Robbins T.W. (2015). Fronto-striatal circuits in response-inhibition: Relevance to addiction. Brain Res..

[43] Brand M., Young K.S., Laier C. (2014). Prefrontal control and Internet addiction: A theoretical model and review of neuropsychological and neuroimaging findings. Front. Hum. Neurosci..

[44] Hong S.-B., Kim J.-W., Choi E.-J., Kim H.-H., Suh J.-E., Kim C.-D., Klauser P., Whittle S., Yűcel M., Pantelis C. (2013). Reduced orbitofrontal cortical thickness in male adolescents with internet addiction. Behav. Brain Funct..

[45] Lin F., Zhou Y., Du Y., Qin L., Zhao Z., Xu J., Lei H. (2012). Abnormal white matter integrity in adolescents with internet addiction disorder: A tract-based spatial statistics study. PLoS ONE.

[46] Oldfield R.C. (1971). The assessment and analysis of handedness: The Edinburgh inventory. Neuropsychologia.

[47] Jang H., Lee J.Y., Lee K.I., Park K.M. (2017). Are there differences in brain morphology according to handedness?. Brain Behav..

[48] Cuzzocreo J.L., Yassa M.A., Verduzco G., Honeycutt N.A., Scott D.J., Bassett S.S. (2009). Effect of handedness on fMRI activation in the medial temporal lobe during an auditory verbal memory task. Hum. Brain Mapp..

[49] Gao Q., Wang J., Yu C., Chen H. (2015). Effect of handedness on brain activity patterns and effective connectivity network during the semantic task of Chinese characters. Sci. Rep..

[50] Jörgens S., Kleiser R., Indefrey P., Seitz R.J. (2007). Handedness and functional MRI-activation patterns in sentence processing. Neuroreport.

[51] Bailey L.M., McMillan L.E., Newman A.J. (2020). A sinister subject: Quantifying handedness-based recruitment biases in current neuroimaging research. Eur. J. Neurosci..

[52] Takeuchi H., Taki Y., Hashizume H., Sassa Y., Nagase T., Nouchi R., Kawashima R. (2011). Failing to deactivate: The association between brain activity during a working memory task and creativity. Neuroimage.

[53] Zamanzadeh V., Ghahramanian A., Rassouli M., Abbaszadeh A., Alavi-Majd H., Nikanfar A.-R. (2015). Design and implementation content validity study: Development of an instrument for measuring patient-centered communication. J. Caring Sci..

[54] Osada H. (2013). Internet addiction in Japanese college students: Is Japanese version of Internet Addiction Test (JIAT) useful as a screening tool. Bull. Senshu Univ. Sch. Hum. Sci..

[55] Raven J.C., Court J. (1938). Raven’s Progressive Matrices.

[56] Rushton J.P., Skuy M., Bons T.A. (2004). Construct validity of Raven’s advanced progressive matrices for African and non-African engineering students in South Africa. Int. J. Sel. Assess..

[57] Beck A.T., Steer R.A., Brown G.K. (1996). Beck Depression Inventory (BDI-II).

[58] Whisman M.A., Richardson E.D. (2015). Normative data on the Beck Depression Inventory–second edition (BDI-II) in college students. J. Clin. Psychol..

[59] Takeuchi H., Taki Y., Hashizume H., Sassa Y., Nagase T., Nouchi R., Kawashima R. (2012). The association between resting functional connectivity and creativity. Cereb. Cortex.

[60] Rodrigues I.B., Adachi J.D., Beattie K.A., MacDermid J.C. (2017). Development and validation of a new tool to measure the facilitators, barriers and preferences to exercise in people with osteoporosis. BMC Musculoskelet. Disord..

[61] Ashburner J., Ridgway G.R. (2013). Symmetric diffeomorphic modeling of longitudinal structural MRI. Front. Neurosci..

[62] Takeuchi H., Taki Y., Hashizume H., Asano K., Asano M., Sassa Y., Yokota S., Kotozaki Y., Nouchi R., Kawashima R. (2015). The impact of television viewing on brain structures: Cross-sectional and longitudinal analyses. Cereb. Cortex.

[63] Smith S.M., Nichols T.E. (2009). Threshold-free cluster enhancement: Addressing problems of smoothing, threshold dependence and localisation in cluster inference. Neuroimage.

[64] Takeuchi H., Taki Y., Asano K., Asano M., Sassa Y., Yokota S., Kotozaki Y., Nouchi R., Kawashima R. (2018). Impact of frequency of internet use on development of brain structures and verbal intelligence: Longitudinal analyses. Hum. Brain Mapp..

[65] Lawshe C.H. (1975). A quantitative approach to content validity. Pers. Psychol..

[66] Fabrigar L.R., Wegener D.T., MacCallum R.C., Strahan E.J. (1999). Evaluating the use of exploratory factor analysis in psychological research. Psychol. Methods.

[67] Bentler P.M. (1992). On the fit of models to covariances and methodology to the Bulletin. Psychol. Bull..

[68] Thompson B. (2006). Foundations of Behavioral Statistics: An Insight-Based Approach.

[69] Gorgich E.A., Moftakhar L., Barfroshan S., Arbabisarjou A. (2018). Evaluation of internet addiction and mental health among medical sciences students in the southeast of Iran. Shiraz E Med. J..

[70] Mousavi S.V. (2020). Prevalence of Internet addiction and the Status of the use of virtual social networks in Iranian Teenagers and Youths in 2018. J. Mil Med..

[71] Alavi S. (2010). Psychometric properties of Young internet addiction test. Int. J. Behav. Sci..

[72] Kuss D.J., Griffiths M.D., Karila L., Billieux J. (2014). Internet addiction: A systematic review of epidemiological research for the last decade. Curr. Pharm. Des..

[73] Ryan T., Chester A., Reece J., Xenos S. (2014). The uses and abuses of Facebook: A review of Facebook addiction. J. Behav. Addict..

[74] Guedes E., Sancassiani F., Carta M.G., Campos C., Machado S., King A.L.S., Nardi A.E. (2016). Internet addiction and excessive social networks use: What about Facebook?. Clin. Pract. Epidemiol. Ment. Health CP EMH.

[75] Lozano Blasco R., Cosculluela C.L., Robres A.Q. (2020). Social network addiction and its impact on anxiety level among university students. Sustainability.

[76] Kuss D.J., Griffiths M.D. (2011). Online social networking and addiction—A review of the psychological literature. Int. J. Environ. Res. Public Health.

[77] Li J.-B., Mo P.K., Lau J.T., Su X.-F., Zhang X., Wu A.M., Mai J.-C., Chen Y.-X. (2018). Online social networking addiction and depression: The results from a large-scale prospective cohort study in Chinese adolescents. J. Behav. Addict..

[78] Kraut R., Patterson M., Lundmark V., Kiesler S., Mukophadhyay T., Scherlis W. (1998). Internet paradox: A social technology that reduces social involvement and psychological well-being?. Am. Psychol..

[79] Raj R. (2020). Internet addiction and three cognitive functions. Int. J. Indian Psychol..

[80] Badre D. (2008). Cognitive control, hierarchy, and the rostro–caudal organization of the frontal lobes. Trends Cogn. Sci..

[81] Badre D., D’esposito M. (2009). Is the rostro-caudal axis of the frontal lobe hierarchical?. Nat. Rev. Neurosci..

[82] Badre D., D’Esposito M. (2007). Functional magnetic resonance imaging evidence for a hierarchical organization of the prefrontal cortex. J. Cogn. Neurosci..

[83] Bahlmann J., Blumenfeld R.S., D’Esposito M. (2015). The rostro-caudal axis of frontal cortex is sensitive to the domain of stimulus information. Cereb. Cortex.

[84] Nee D.E., D’Esposito M. (2016). The hierarchical organization of the lateral prefrontal cortex. Elife.

[85] Owsley C., McGwin G., McNeal S.F. (2003). Impact of impulsiveness, venturesomeness, and empathy on driving by older adults. J. Saf. Res..

[86] Carlson S.R., Johnson S.C., Jacobs P.C. (2010). Disinhibited characteristics and binge drinking among university student drinkers. Addict. Behav..

[87] Galimberti E., Martoni R.M., Cavallini M.C., Erzegovesi S., Bellodi L. (2012). Motor inhibition and cognitive flexibility in eating disorder subtypes. Prog. Neuro-Psychopharmacol. Biol. Psychiatry.

[88] Birkley E.L., Smith G.T. (2011). Recent advances in understanding the personality underpinnings of impulsive behavior and their role in risk for addictive behaviors. Curr. Drug Abus. Rev..

